# Genomic analysis and relatedness of P2-like phages of the *Burkholderia cepacia *complex

**DOI:** 10.1186/1471-2164-11-599

**Published:** 2010-10-25

**Authors:** Karlene H Lynch, Paul Stothard, Jonathan J Dennis

**Affiliations:** 1 CW405 Biological Sciences Building, Department of Biological Sciences, University of Alberta, Edmonton, Alberta, T6G 2E9, Canada; 2 1400 College Plaza, Department of Agricultural, Food and Nutritional Science, University of Alberta, Edmonton, Alberta, T6G 2C8, Canada

## Abstract

**Background:**

The *Burkholderia cepacia *complex (BCC) is comprised of at least seventeen Gram-negative species that cause infections in cystic fibrosis patients. Because BCC bacteria are broadly antibiotic resistant, phage therapy is currently being investigated as a possible alternative treatment for these infections. The purpose of our study was to sequence and characterize three novel BCC-specific phages: KS5 (vB_BceM-KS5 or vB_BmuZ-ATCC 17616), KS14 (vB_BceM-KS14) and KL3 (vB_BamM-KL3 or vB_BceZ-CEP511).

**Results:**

KS5, KS14 and KL3 are myoviruses with the A1 morphotype. The genomes of these phages are between 32317 and 40555 base pairs in length and are predicted to encode between 44 and 52 proteins. These phages have over 50% of their proteins in common with enterobacteria phage P2 and so can be classified as members of the *Peduovirinae *subfamily and the "P2-like viruses" genus. The BCC phage proteins similar to those encoded by P2 are predominantly structural components involved in virion morphogenesis. As prophages, KS5 and KL3 integrate into an AMP nucleosidase gene and a threonine tRNA gene, respectively. Unlike other P2-like viruses, the KS14 prophage is maintained as a plasmid. The P2 *E+E' *translational frameshift site is conserved among these three phages and so they are predicted to use frameshifting for expression of two of their tail proteins. The *lysBC *genes of KS14 and KL3 are similar to those of P2, but in KS5 the organization of these genes suggests that they may have been acquired via horizontal transfer from a phage similar to λ. KS5 contains two sequence elements that are unique among these three phages: an IS*Bmu*2-like insertion sequence and a reverse transcriptase gene. KL3 encodes an EcoRII-C endonuclease/methylase pair and Vsr endonuclease that are predicted to function during the lytic cycle to cleave non-self DNA, protect the phage genome and repair methylation-induced mutations.

**Conclusions:**

KS5, KS14 and KL3 are the first BCC-specific phages to be identified as P2-like. As KS14 has previously been shown to be active against *Burkholderia cenocepacia in vivo*, genomic characterization of these phages is a crucial first step in the development of these and similar phages for clinical use against the BCC.

## Background

The *Burkholderia cepacia *complex (BCC) is a group of at least seventeen species of Gram-negative opportunistic pathogens. Although these organisms can infect patients with a broad range of chronic conditions, the majority of infections occur in those with cystic fibrosis (CF) [1,2]. Because the lungs of these individuals contain thick mucus that cannot be cleared by the mucociliary escalator, they are susceptible to pulmonary infections by microorganisms such as *Pseudomonas*, *Staphylococcus*, *Haemophilus *and *Burkholderia *[3,4]. The prevalence of BCC infection in American CF patients was 3.1% in 2005 [5]. Although this prevalence is low compared to that of *Pseudomonas aeruginosa *(56.1% in 2005), there are three reasons why the BCC is a serious problem for the CF population [5]. First, BCC bacteria cause severe and potentially fatal respiratory infections. When compared to patients infected with *Pseudomonas*, those with BCC infections have reduced lung function and, depending on the species present, increased mortality [6,7]. In approximately 20% of cases, these individuals develop a rapidly fatal condition called 'cepacia syndrome,' which is characterized by lung abscesses and septicemia [2,8]. Second, BCC bacteria can spread from person-to-person. It has been shown that at least five BCC species can be transmitted in this manner: *Burkholderia cepacia*, *Burkholderia multivorans*, *Burkholderia cenocepacia*, *Burkholderia dolosa *and *Burkholderia contaminans *[9-11]. Because of the potential for these organisms to spread among a susceptible population, BCC culture-positive patients are isolated from other individuals with CF, a measure that has serious social and psychological implications [12,13]. Finally, BCC bacteria are resistant to most antibiotics. These species have a variety of resistance mechanisms including β-lactamases, efflux pumps and biofilm formation [14-16]. The most effective anti-BCC antibiotics - ceftazidime, meropenem and minocycline - only inhibit between 23-38% of clinical isolates [17].

Because conventional antibiotics are largely ineffective against the BCC, phage therapy is being explored as a possible alternative treatment. Phage therapy is the clinical administration of bacteriophages (or phages) to prevent and/or to treat bacterial infections [18]. Although phages have been used therapeutically for almost a century, this treatment fell out of favor in North America and Western Europe when penicillin and other chemical antibiotics became widely available in the 1940s [18]. However, there has been renewed interest in this field following the emergence of multidrug resistant bacteria such as those of the BCC [18]. Three recent studies have shown that phages are active against the BCC *in vivo*. Seed and Dennis showed that treatment of *B. cenocepacia*-infected *Galleria mellonella *larvae with phages KS14, KS4-M or KS12 increased survival 48 hours post-infection, even when treatment with the latter two phages was delayed for 6 to 12 hours [19]. Carmody et al. showed that intraperitoneal administration of phage BcepIL02 to *B. cenocepacia*-infected mice decreased bacterial density in the lungs and led to decreased expression of the pro-inflammatory cytokines MIP-2 and TNF-α [20]. Lynch et al. published the first description of an engineered BCC phage and showed that this mutant (a repressor knockout of phage KS9) was able to increase survival of *B. cenocepacia*-infected *G. mellonella *48 hours post-infection [21].

Before a phage can be safely used clinically, its complete genome sequence must be determined to assess whether the phage is obligately lytic or temperate, and to determine by homology whether the phage genome encodes any putative virulence factors. This report describes the genome sequence of three novel BCC phages and their relatedness to enterobacteria phage P2. P2 is a temperate myovirus that was isolated from *E. coli *strain Li by Bertani in 1951 [22]. P2 has recently been classified as part of a novel subfamily, placing it in the order *Caudovirales*, family *Myoviridae*, subfamily *Peduovirinae *and genus "P2-like viruses" [23]. This genus includes phages P2, Wϕ, 186 and PsP3 of enterobacteria, L-413C of *Yersinia*, Fels-2 and SopEϕ of *Salmonella*, ϕ-MhaA1-PHL101 of *Mannheimia*, ϕCTX of *Pseudomonas*, RSA1 of *Ralstonia*, ϕE202 of *Burkholderia thailandensis *and ϕ52237 and ϕE12-2 of *Burkholderia pseudomallei *[23]. Based on sequence analysis, it is proposed that the BCC-specific phages KS5 (vB_BceM-KS5 or vB_BmuZ-ATCC 17616), KS14 (vB_BceM-KS14) and KL3 (vB_BamM-KL3 or vB_BceZ-CEP511) should also be classified as part of this genus [24].

## Results and Discussion

### Isolation, host range and morphology

Seed and Dennis isolated KS5 from an extract of onion soil plated on *B. cenocepacia *K56-2 [25]. This phage produces clear plaques on K56-2 with a diameter of 0.5-1.0 mm [25]. Previously, KS5 was tested for its ability to form plaques on K56-2 LPS mutants and it was determined that it could efficiently lyse wildtype K56-2 (EOP = 1), XOA7 (*waaL*::pGPΩTp, EOP = 0.8), XOA15 (*wabR*::pGPΩTp, EOP = 1.3), XOA17 (*wabS*::pGPΩTp, EOP = 1.1) and RSF19 (*wbxE*::pGPΩTp, EOP = 0.5), but not XOA8 (*wabO*::pGPΩTp) or CCB1 (*waaC*::pGPΩTp) [21,26,27]. Based on these results, it was predicted that KS5 uses the K56-2 LPS as a receptor and that it binds within the core region [21]. KS5 has a relatively wide host range compared to many BCC phages, infecting *B. multivorans *C5393 and *B. cenocepacia *715J, J2315, K56-2, C6433 and C5424 [25].

KS14 was isolated from an extract of *Dracaena* sp. soil plated on *B. multivorans * C5393 [19]. Both BCC phages and bacteria have been recovered from the *Dracaena *rhizosphere [19,28]. On C5393, KS14 forms small clear plaques 0.5-1.0 mm in diameter, similar to the morphology of KS5 on K56-2. The host range of KS14 includes *B. multivorans *C5393 and C5274, *B. cenocepacia *715J, C6433, C5424 and PC184, *B. dolosa *LMG 21443 and *Burkholderia ambifaria *LMG 17828 [19].

KL3 was isolated from a single plaque on a lawn of *B. cenocepacia *CEP511, an Australian CF epidemic isolate [29]. Phage induction from CEP511 was stochastic, as treatment with inducing agents such as UV or mitomycin C was not necessary. On LMG 17828, KL3 forms small turbid plaques 0.5-1.0 mm in diameter. KL3 has a narrow host range, infecting *B. ambifaria *LMG 17828.

**Figure 1:**
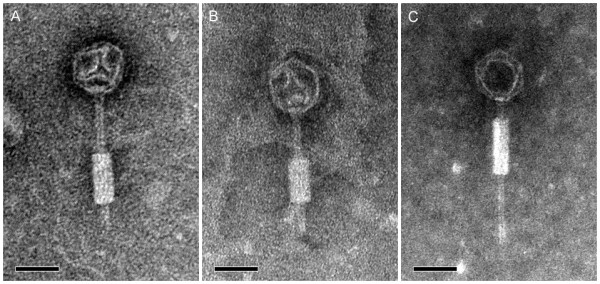
**Transmission electron micrographs of KS5 (A), KS14 (B) and KL3 (C)**. Phages were stained with 2% phosphotungstic acid and viewed at 140,000-fold magnification. Scale bars represent 50 nm.

Electron microscopy of KS5, KS14 and KL3 indicates that these phages belong to the family *Myoviridae *(Figure 1). These three phages exhibit the A1 morphotype, with icosahedral capsids and contractile tails [30]. KS5, KS14 and KL3 have similarly sized capsids, each 65 nm in diameter (Figure 1). In contrast, their tails vary in length: 140 nm for KS14, 150 nm for KS5 and 160 nm for KL3 (Figure 1). These sizes correspond to the length of the tail tape measure protein for each of these three phages: KS14 gp12 is 842 amino acids (aa) in length, KS5 gp15 is 920 aa and KL3 gp17 is 1075 aa (Tables 1, 2 and 3).

**Table 1:** KS5 genome annotation

Gene	Start	End	Putative function	Strand	Predicted RBS and start codon	Length (no. of aa residues)	Closest relative (excluding ATCC 17616)	Alignment region (no. of aa residues)	% ID	Source	**GenBank accession no**.	ATCC 17616 locus tag	**ATCC 17616 GenBank accession no**.
*1*	108	815	integrase	-	AGCAACAAGcacaaggcaTTG	235	integrase family protein	134-368/368	87	*Burkholderia thailandensis *MSMB43	ZP_02468407.1	BMULJ_03640	YP_001948048.1
*2*	2142	4937	zinc finger CHC2-family protein	-	GAGCAACAGcaataacgATG	931	conserved hypothetical protein	1-931/931	96	*Burkholderia multivorans *CGD1	ZP_03587581.1	BMULJ_03641	YP_001948049.1
*3*	4940	5200	unknown	-	GGGGGAAGccgcATG	86	conserved hypothetical protein	1-86/86	91	*Burkholderia multivorans *CGD1	ZP_03587582.1	BMULJ_03642	YP_001948050.1
*4*	5197	5556	unknown	-	GGGGGTGAtgtgATG	119	conserved hypothetical protein	1-119/119	97	*Burkholderia multivorans *CGD1	ZP_03587583.1	BMULJ_03643	YP_001948051.1
*5*	5561	5755	membrane protein	-	GGAGccaaaccATG	64	putative phage-encoded membrane protein	1-64/64	78	*Burkholderia ambifaria *MEX-5	ZP_02905725.1	BMULJ_03644	YP_001948052.1
*6*	5798	6001	unknown	-	GGATGcactgaccgATG	67	conserved hypothetical protein	1-67/67	92	*Burkholderia multivorans *CGD1	ZP_03587585.1	BMULJ_03645	YP_001948053.1
*7*	6005	6199	unknown	-	GGAGAGActcATG	64	conserved hypothetical protein	1-64/64	98	*Burkholderia multivorans *CGD1	ZP_03587586.1	BMULJ_03646	YP_001948054.1
*8*	6289	6537	transcriptional activator (Ogr)	-	GTAGGAGccccgaATG	82	transcriptional activator Ogr/delta	1-82/82	91	*Burkholderia cenocepacia *MC0-3	YP_001763475.1	BMULJ_03647	YP_001948055.1
*9*	6547	6825	DNA binding protein	-	GGGCGttgagtcATG	92	putative phage DNA-binding protein	1-92/92	98	*Burkholderia ambifaria *MEX-5	ZP_02905729.1	BMULJ_03648	YP_001948056.1
*10*	6829	7065	unknown	-	GAAGGGAAGtataccgtcATG	78	putative bacteriophage protein	1-77/78	80	*Burkholderia sp*. CCGE1001	ZP_06292840.1	BMULJ_03649	YP_001948057.1
*11*	7121	7603	repressor	+	GATAATACAcaccgatcgGTG	160	putative phage DNA-binding protein	12-166/167	79	*Burkholderia pseudomallei *K96243	YP_106769.1	BMULJ_03650	YP_001948058.1
*12*	7660	8406	membrane protein	+	AGGGAAttcaATG	248	putative phage-encoded membrane protein	1-241/249	43	*Burkholderia pseudomallei *K96243	YP_106770.1	BMULJ_03651	YP_001948059.1
	8971	8975	direct repeat flanking IS*Bmu*23										
IS*Bmu*23	8976	10185	IS*Bmu*23 insertion sequence										
	8976	8991	IS*Bmu*23 inverted repeat										
	9063	10055	IS*Bmu*23 transposase	+	GGAACGGAcccacgacgATG	330	transposase IS4 family protein	1-330/330	100	*Burkholderia sp*. Ch1-1	ZP_06846513.1	BMULJ_03652	YP_001948060.1
	10170	10185	IS*Bmu*23 inverted repeat										
	10185	10189	direct repeat flanking IS*Bmu*23										
*13*	10359	11510	tail protein (D)	-	AAGGAGGcgatctcgctATG	383	phage late control gene D protein	1-379/382	96	*Burkholderia multivorans *CGD1	ZP_03587594.1	BMULJ_03653	YP_001948061.1
*14*	11507	11935	tail protein (U)	-	GAAGGAGGGAttgtcATG	142	bacteriophage gpU	1-142/142	95	*Burkholderia multivorans *CGD1	ZP_03587595.1	BMULJ_03654	YP_001948062.1
*15*	11949	14711	tail tape measure protein (T)	-	GAGCGAGGcgacgaATG	920	putative phage-related tail transmembrane protein	1-919/919	91	*Burkholderia cenocepacia *MC0-3	YP_001763483.1	BMULJ_03655	YP_001948063.1
*16*	14827	15138	tail protein (E)	-	AGAGGAAccatacgATG	103	phage tail protein E	1-103/103	97	*Burkholderia multivorans *CGD1	ZP_03587598.1	BMULJ_03657	YP_001948065.1
*17*	14708	15138	tail protein (E+E')	-	AGAGGAAccatacgATG	143	phage tail protein E	1-87/103	97	*Burkholderia multivorans *CGD1	ZP_03587598.1	BMULJ_03656 BMULJ_03657	YP_001948064.1 YP_001948065.1
*18*	15171	15680	tail tube protein (FII)	-	AGGGAAAcgcaATG	169	phage major tail tube protein	1-169/169	94	*Burkholderia multivorans *CGD1	ZP_03587599.1	BMULJ_03658	YP_001948066.1
*19*	15710	16882	tail sheath protein (FI)	-	GGGAGAttgcATG	390	tail sheath protein	1-390/390	94	*Burkholderia cenocepacia *MC0-3	YP_001763487.1	BMULJ_03659	YP_001948067.1
*20*	16993	17742	N-4/N-6 DNA methylase	-	GAGGGAAtcgccccATG	249	DNA methylase N-4/N-6 domain protein	1-249/249	89	*Burkholderia ambifaria *MEX-5	ZP_02905740.1	BMULJ_03660	YP_001948068.1
*21*	17720	17902	Com translational regulator	-	AAGCAGGAAtcacccgATG	60	hypothetical protein Bcenmc03_0187	1-60/60	85	*Burkholderia cenocepacia *MC0-3	YP_001763489.1	BMULJ_03661	YP_001948069.1
*22*	18049	18927	tail fiber assembly protein	-	GAGACACAcctATG	292	gp31, bacteriophage-acquired protein	1-272/278	89	*Burkholderia multivorans *CGD1	ZP_03587603.1	BMULJ_03662	YP_001948070.1
*23*	18937	20547	tail fiber protein	-	GGATAcctgaacATG	536	bacteriophage protein	1-536/536	99	*Burkholderia multivorans *CGD1	ZP_03587604.1	BMULJ_03663	YP_001948071.1
*24*	20550	21104	baseplate assembly protein (I)	-	GGGGTGGccgATG	184	ZP_03587605.1	1-184/184	92	*Burkholderia multivorans *CGD1	ZP_03587605.1	BMULJ_03664	YP_001948072.1
*25*	21097	22002	baseplate assembly protein (J)	-	GAGGCAcggcATG	301	ZP_03587606.1	1-301/301	94	*Burkholderia multivorans *CGD1	ZP_03587606.1	BMULJ_03665	YP_001948073.1
*26*	21999	22376	ZP_03587607.1	-	GAAGGGGcacggATG	125	baseplate assembly protein W (GpW)	1-125/125	89	*Burkholderia multivorans *CGD1	ZP_03587607.1	BMULJ_03666	YP_001948074.1
*27*	22373	23005	baseplate assembly protein (V)	-	GCGGCAtccttgccgcATG	210	YP_001763496.1	1-137/234	78	*Burkholderia cenocepacia *MC0-3	YP_001763496.1	BMULJ_03667	YP_001948075.1
*28*	23206	25086	exonuclease (Old)	-	AAGTGGGGAccaactATG	626	ATP-dependent endonuclease	1-625/626	72	*Cupriavidus metallidurans *CH34	YP_586772.1	BMULJ_03668	YP_001948076.1
*29*	25269	25718	tail completion protein (S)	-	GGGGAcgtgATG	149	phage virion morphogenesis protein	1-148/149	89	*Burkholderia multivorans *CGD1	ZP_03587610.1	BMULJ_03669	YP_001948077.1
*30*	25718	26128	tail completion protein (R)	-	AGGAGGcgccGTG	136	P2 phage tail completion protein R (GpR)	1-136/136	96	*Burkholderia multivorans *CGD1	ZP_03587611.1	BMULJ_03670	YP_001948078.1
*31*	26172	26366	Rz1	-	AAGGAGGttccggtttATG	64	Ribonuclease, Rne/Rng family	15-48/928	47	*Propionibacterium freudenreichii *subsp. *shermanii *CIRM-BIA1	YP_003687809.1	none	
*32*	26125	26616	Rz	-	GGGTGGccgcATG	163	conserved hypothetical protein	1-163/163	85	*Burkholderia ambifaria *MEX-5	ZP_02905751.1	BMULJ_03671	YP_001948079.1
*33*	26613	27413	endolysin	-	GGGGGcgccATG	266	peptidoglycan binding domain-containing protein	1-266/266	90	*Burkholderia cenocepacia *MC0-3	YP_001763501.1	BMULJ_03672	YP_001948080.1
*34*	27406	27726	holin	-	AAGGGGAGGGAcaagtgATG	106	protein of unknown function DUF754	1-106/106	88	*Burkholderia ambifaria *MEX-5	ZP_02905753.1	BMULJ_03673	YP_001948081.1
*35*	27726	28100	putative antiholin	-	ATGGGActgagaATG	124	phage-related transmembrane protein	1-124/124	96	*Burkholderia multivorans *CGD1	ZP_03587615.1	BMULJ_03674	YP_001948082.1
*36*	28103	28315	tail protein (X)	-	AGGGAGctgtcctgATG	70	tail X family protein	1-70/70	94	*Burkholderia cenocepacia *MC0-3	YP_001763504.1	BMULJ_03675	YP_001948083.1
*37*	28315	28557	unknown	-	GTGGAGctcatctgATG	80	conserved hypothetical protein	1-80/80	72	*Burkholderia multivorans *CGD1	ZP_03587617.1	BMULJ_03676	YP_001948084.1
*38*	28557	29033	capsid completion protein (L)	-	AACGTGACGAAcccgaccATG	158	head completion protein	1-160/160	85	*Burkholderia ambifaria *MEX-5	ZP_02905755.1	BMULJ_03677	YP_001948085.1
*39*	29138	29824	terminase endonuclease subunit (M)	-	GGGTGGcgcATG	228	terminase	1-228/228	93	*Burkholderia multivorans *CGD1	ZP_03587619.1	BMULJ_03678	YP_001948086.1
*40*	29821	30846	capsid protein (N)	-	AAACGGAGAAtccATG	341	phage major capsid protein, P2 family	1-339/339	77	*Burkholderia ambifaria *MEX-5	ZP_02905757.1	BMULJ_03679	YP_001948087.1
*41*	30884	31705	capsid scaffolding protein (O)	-	AGAGGtttcgcacATG	273	phage capsid scaffolding protein GpO	1-273/273	95	*Burkholderia multivorans *CGD1	ZP_03587621.1	BMULJ_03680	YP_001948088.1
*42*	31855	33621	terminase ATPase subunit (P)	+	GGTAGccttgctgcATG	588	putative ATPase subunit of terminase (gpP-like)	1-583/583	92	*Burkholderia multivorans *CGD1	ZP_03587622.1	BMULJ_03681	YP_001948089.1
*43*	33621	34673	portal vertex protein (Q)	+	ATGGAGAttttctgATG	350	phage portal protein, pbsx family	1-348/349	92	*Burkholderia multivorans *CGD1	ZP_03587623.1	BMULJ_03682	YP_001948090.1
*44*	35144	36163	reverse transcriptase	-	GAATGGAtttccgaaaATG	339	putative reverse transcriptase	2-285/292	42	*Sideroxydans lithotrophicus *ES-1	YP_003522714.1	BMULJ_03683	YP_001948091.1
*45*	36120	36443	transcriptional regulator	-	GAAGGAGttgcatATG	107	transcriptional regulator	1-97/97	52	*Acinetobacter baumannii *ACICU	YP_001840883.1	BMULJ_03684	YP_001948092.1

Abbreviations: RBS, ribosome-binding site; aa, amino acid, % ID, percent identity. The P2 proteins that are similar to KS5 proteins based on CoreGenes analysis are shown in brackets in the putative function column. Excluding genes *17 *and *31*, annotations were based on those of the *B. multivorans *ATCC 17616 chromosome 2 sequence (NC_010805.1; BMULJ_03640 - BMULJ_03684, bp 477496-514731).

**Table 2:** KS14 genome annotation

Gene	Start	End	Putative function	Strand	Predicted RBS and start codon	Length (no. of aa residues)	Closest relative	Alignment region (no. of aa residues)	% ID	Source	**GenBank accession no**.
*1*	1	261	unknown	-	GAGGCGAggcATG	86	hypothetical protein BB1680	18-96/101	35	*Bordetella bronchiseptica *RB50	NP_888225.1
*2*	270	3041	zinc finger CHC2-family protein	-	GCGATTCTGAaaaATG	923	hypothetical protein RPRSA1_gp47	1-933/934	65	*Ralstonia *phage phiRSA1	YP_001165296.1
*3*	3122	3511	unknown	-	GAGGGAccgaaccATG	129	hypothetical protein Csal_1360	9-103/130	33	*Chromohalobacter salexigens *DSM 3043	YP_573414.1
*4*	3857	4036	unknown	-	GAAAAcaccATG	59	hypothetical protein PC1_2629	21-62/63	45	*Pectobacterium carotovorum *subsp. carotovorum PC1	YP_003018195.1
*5*	4142	4849	repressor	+	AAGGcccaatATG	235	hypothetical protein GCWU000324_01220	13-220/226	33	*Kingella oralis *ATCC 51147	ZP_04601747.1
*6*	4809	5486	serine recombinase	-	GAAGGCGAtacaagaaaATG	225	resolvase domain-containing protein	4-194/195	57	*Shewanella *sp. W3-18-1	YP_965429.1
*7*	5479	5790	unknown	-	GAGGCGGGcgagctATG	103	hypothetical protein BuboB_03089	9-106/114	45	*Burkholderia ubonensis *Bu	ZP_02376681.1
*8*	5787	6368	unknown	-	GACTACAGGcgaccATG	193	hypothetical protein pRALTA_0144	25-180/255	48	*Cupriavidus taiwanensis*	YP_001796036.1
*9*	6506	7054	unknown	+	AGGAAAGAAAAcggtcgtTTG	182	hypothetical protein Ajs_3318	20-138/138	31	*Acidovorax sp*. JS42	YP_987516.1
*10*	7093	8130	tail protein (D)	-	AAAAAagaATG	345	fels-2 prophage protein	25-366/366	65	*Burkholderia oklahomensis *EO147	ZP_02353972.1
*11*	8127	8558	tail protein (U)	-	GGAGGAAAGAAAcgATG	143	bacteriophage tail-related protein	1-133/141	64	*Burkholderia oklahomensis *EO147	ZP_02353973.1
*12*	8574	11102	tail tape measure protein (T)	-	GTATGGAAGcgaATG	842	phage tail tape measure protein, TP901 family	5-918/924	39	*Pantoea sp*. At-9 b	ZP_05730476.1
*13*	11251	11535	tail protein (E)	-	AGAGAAAgaaATG	94	hypothetical protein BPSL0148	12-103/114	72	*Burkholderia pseudomallei *K96243	YP_106776.1
*14*	11099	11535	tail protein (E+E')	-	AGAGAAAgaaATG	145	gpE+E'	3-142/142	49	Enterobacteria phage P2	NP_046780.1
*15*	11623	12138	tail tube protein (FII)	-	AGGGAGtaaATG	171	phage major tail tube protein	1-169/169	66	*Burkholderia sp*. CCGE1001	ZP_06292830.1
*16*	12150	13325	tail sheath protein (FI)	-	AAACAGGAAttcagATG	391	putative phage major tail sheath protein	1-390/390	72	*Burkholderia cenocepacia *J2315	YP_002229261.1
*17*	13387	14007	tail fiber assembly protein (G)	-	GAAGGGAAccgaccATG	206	gp31, bacteriophage-acquired protein	1-189, 196-266/278	66, 54	*Burkholderia multivorans *CGD1	ZP_03587603.1
*18*	14026	15534	tail fiber protein	-	ACGGATAtctgacctATG	502	hypothetical protein BuboB_27067	3-534/534	55	*Burkholderia ubonensis *Bu	ZP_02381413.1
*19*	15538	16080	baseplate assembly protein (I)	-	GGGGGCAtttacgaaaATG	180	phage-related tail protein	1-179/180	73	*Burkholderia ubonensis *Bu	ZP_02381412.1
*20*	16073	16987	baseplate assembly protein (J)	-	AACGGGGGTGcggcATG	304	phage baseplate assembly protein	1-303/304	74	*Burkholderia ubonensis *Bu	ZP_02381411.1
*21*	16984	17346	baseplate assembly protein (W)	-	GTGAGCGCAccgcaATG	120	phage baseplate assembly protein	1-117/117	66	*Burkholderia thailandensis *MSMB43	ZP_02466378.1
*22*	17343	18002	baseplate assembly protein (V)	-	AACATGGAggcATG	219	bacteriophage baseplate assembly protein V	1-227/227	57	*Burkholderia ubonensis *Bu	ZP_02381409.1
*23*	18087	18545	tail completion protein (S)	-	GATCCGGCGGcgcaATG	152	phage virion morphogenesis protein	1-153/155	66	*Burkholderia sp*. CCGE1001	ZP_06292822.1
*24*	18533	18985	tail completion protein (R)	-	ACCGcccccgaccATG	150	P2 phage tail completion R family protein	1-136/140	54	*Burkholderia sp*. CCGE1001	ZP_06292821.1
*25*	18978	19256	Rz1 (LysC)	-	GAGGCGttgaaacATG	92	hypothetical phage protein	1-83/91	59	*Burkholderia pseudomallei *1655	ZP_04890536.1
*26*	19102	19554	Rz (LysB)	-	GAGAAGGcggccgcATG	150	putative phage-encoded lipoprotein	19-142/142	44	*Burkholderia glumae *BGR1	YP_002910045.1
*27*	19551	20357	endolysin	-	GCGGAGtgaATG	268	putative phage-encoded peptidoglycan binding protein	5-268/268	66	*Burkholderia ubonensis *Bu	ZP_02376668.1
*28*	20354	20620	holin	-	GAAAGGGctgacccATG	88	protein of unknown function DUF754	1-88/88	61	*Burkholderia sp*. CCGE1001	ZP_06292819.1
*29*	20624	20965	putative antiholin	-	GGAGtcgccaacATG	113	hypothetical protein BuboB_26997	1-113/114	74	*Burkholderia ubonensis *Bu	ZP_02381399.1
*30*	20980	21186	tail protein (X)	-	GATCGAGctgatctgATG	68	putative phage tail protein	1-67/67	61	*Erwinia tasmaniensis *Et1/99	YP_001906519.1
*31*	21186	21677	capsid completion protein (L)	-	AGAGctgaaaccATG	163	fels-2 prophage protein	31-172/172	55	*Burkholderia thailandensis *E264	YP_439544.1
*32*	21779	22465	terminase, endonuclease subunit (M)	-	AACGGAGGcatgacgcgATG	228	bacteriophage terminase, endonuclease subunit	3-220/229	59	*Burkholderia oklahomensis *EO147	ZP_02360025.1
*33*	22481	23497	capsid protein (N)	-	GGAGAAcacaccacATG	338	bacteriophage protein	1-338/338	68	*Ralstonia solanacearum *GMI1000	NP_520058.1
*34*	23540	24577	capsid scaffolding protein (O)	-	GGAGAcctaacaATG	345	capsid scaffolding	4-349/349	50	*Burkholderia sp*. CCGE1001	ZP_06292813.1
*35*	24693	26507	terminase ATPase subunit (P)	+	GGGTACAcataggcgggcGTG	604	protein of unknown function DUF264	13-586/588	75	*Burkholderia sp*. CCGE1001	ZP_06292812.1
*36*	26507	27559	portal vertex protein (Q)	+	ATGGAGttctcttaATG	350	putative phage portal vertex protein	1-347/351	70	*Burkholderia pseudomallei *7894	ZP_02487524.1
*37*	27740	28495	replication initiation	-	AGGGGAAGcgtcccaATG	251	initiator RepB protein	16-251/251	73	*Ralstonia pickettii *12J	YP_001901323.1
*38*	28834	29010	unknown	+	GTGAGGGGcaacaaGTG	58	none				
*39*	29150	29695	unknown	+	GTGATGCACGAccgcccgaATG	181	flagellar hook-associated protein FlgK	257-334/672	29	*Acidovorax ebreus *TPSY	YP_002554543.1
*40*	30436	31086	DNA partitioning	+	GGAGCATGcgaaATG	216	ParA family protein, putative	1-211/217	69	*Burkholderia thailandensis *E264	YP_439556.1
*41*	31127	31438	unknown	+	AGCGAGGtaatagcaaaATG	103	hypothetical protein BuboB_03094	1-73/88	49	*Burkholderia ubonensis *Bu	ZP_02376682.1
*42*	31435	31581	unknown	+	AAAGAGGGggcATG	48	none				
*43*	31603	31821	unknown	+	GAAAAGGGGAAttgaATG	72	hypothetical protein SMR0083	10-63/63	57	*Serratia marcescens*	NP_941157.1
*44*	31909	32217	unknown	-	GGAGTGAtgtttATG	102	hypothetical protein BuboB_03104	1-69/84	73	*Burkholderia ubonensis *Bu	ZP_02376684.1

Abbreviations: RBS, ribosome-binding site; aa, amino acid; % ID, percent identity. The P2 proteins that are similar to KS14 proteins based on CoreGenes analysis are shown in brackets in the putative function column.

**Table 3:** KL3 genome annotation

Gene	Start	End	Putative function	Strand	Predicted RBS and start codon	Length (no. of aa residues)	Closest relative	Alignment region (no. of aa residues)	% ID	Source	**GenBank accession no**.
*1*	122	1150	integrase	-	GGCGCAGtgtgATG	342	integrase family protein	1-342/342	98	*Burkholderia ambifaria *MEX-5	ZP_02905720.1
*2*	1150	1416	unknown	-	GAAAAtcaccATG	88	hypothetical protein BamMEX5DRAFT_1075	1-88/88	97	*Burkholderia ambifaria *MEX-5	ZP_02905721.1
*3*	2096	4891	zinc finger CHC2-family protein	-	AACAGCAAtaacgATG	931	zinc finger CHC2-family protein	1-931/931	95	*Burkholderia ambifaria *MEX-5	ZP_02905722.1
*4*	4894	5142	unknown	-	GGAGGcgcagcagcATG	82	conserved hypothetical protein	1-82/84	91	*Burkholderia ambifaria *MEX-5	ZP_02905723.1
*5*	5139	5501	unknown	-	GCGGGGctgacacgATG	120	conserved hypothetical protein	1-120/120	97	*Burkholderia ambifaria *MEX-5	ZP_02905724.1
*6*	5506	5700	membrane protein	-	GGAAccacaccATG	64	putative phage-encoded membrane protein	1-64/64	93	*Burkholderia ambifaria *MEX-5	ZP_02905725.1
*7*	5744	5938	unknown	-	GCACTGAtccgATG	64	hypothetical protein bglu_1 g03740	1-64/64	95	*Burkholderia glumae *BGR1	YP_002910278.1
*8*	5943	6155	unknown	-	GAAAAAAGGAGAtcagcATG	70	conserved hypothetical protein	1-70/70	72	*Burkholderia sp*. CCGE1001	ZP_06292843.1
*9*	6243	6491	transcriptional activator (Ogr)	-	GGAGTAAGccgaaATG	82	putative phage transcriptional activator Ogr/Delta	1-82/82	92	*Burkholderia glumae *BGR1	YP_002910024.1
*10*	6479	6682	unknown	-	AATGAGTAGctcctacgATG	67	hypothetical protein BoklE_00724	1-67/67	85	*Burkholderia oklahomensis *EO147	ZP_02353966.1
*11*	6722	6919	unknown	-	GAGGAGcccgcATG	65	hypothetical protein BoklE_00729	1-65/65	87	*Burkholderia oklahomensis *EO147	ZP_02353967.1
*12*	6934	7143	unknown	-	AAAGTATAccgaccATG	69	hypothetical protein BoklE_00734	1-62/71	87	*Burkholderia oklahomensis *EO147	ZP_02353968.1
*13*	7193	7732	repressor	+	GGTAAGGctagtgtaATG	179	hypothetical protein BCAL0086	1-163/163	62	*Burkholderia cenocepacia *J2315	YP_002229252.1
*14*	7904	8179	unknown	+	GAGGGAccagaagaATG	91	hypothetical protein BuboB_27112	1-91/98	50	*Burkholderia ubonensis *Bu	ZP_02381422.1
*15*	8238	9329	tail protein (D)	-	GGACGCGGAGccgaaggcATG	363	fels-2 prophage protein	19-366/366	81	*Burkholderia oklahomensis *EO147	ZP_02353972.1
*16*	9326	9784	tail protein (U)	-	ACGGAGGAtctgtcccATG	152	bacteriophage tail-related protein	1-133/141	66	*Burkholderia oklahomensis *EO147	ZP_02353973.1
*17*	9806	13033	tail tape measure protein (T)	-	GAAGCGGAcacgagtaacgATG	1075	hypothetical protein bglu_1 g01240	1-1079/1079	59	*Burkholderia glumae *BGR1	YP_002910030.1
*18*	13158	13508	tail protein (E)	-	AGGACACGcaacatATG	116	gpE+E'	1-114/114	76	*Burkholderia pseudomallei *112	ZP_02501899.1
*19*	13036	13508	tail protein (E+E')	-	AGGACACGcaacatATG	157	gpE+E'	1-100/114	74	*Burkholderia pseudomallei *112	ZP_02501899.1
*20*	13578	14087	tail tube protein (FII)	-	AGGAGtcacacacATG	169	phage major tail tube protein	1-169/169	74	*Burkholderia cenocepacia *J2315	YP_002229260.1
*21*	14103	15275	tail sheath protein (FI)	-	AGGAGctgcacaccATG	390	phage tail sheath protein	1-390/390	84	*Burkholderia pseudomallei *1655	ZP_04890547.1
*22*	15328	15951	tail fiber assembly protein	-	ACGGAcctcgaaacATG	207	tail fiber assembly protein from lambdoid prophage e14	1-190/209	85	*Burkholderia ubonensis *Bu	ZP_02381414.1
*23*	15969	18632	tail fiber protein	-	GGATAcctgaacATG	887	putative phage tail protein	1-883/883	71	*Burkholderia cenocepacia *J2315	YP_002229263.1
*24*	18635	19189	baseplate assembly protein (I)	-	GATGGCGGGGtcgcggATG	184	phage-related tail protein	1-183/184	84	*Burkholderia pseudomallei *7894	ZP_02487653.1
*25*	19182	20087	baseplate assembly protein (J)	-	GAACGGAGtcggcgcATG	301	baseplate J-like protein	1-301/301	90	*Burkholderia thailandensis *E264	YP_439531.1
*26*	20084	20446	baseplate assembly protein (W)	-	GGAGCGGtgcATG	120	phage baseplate assembly protein	1-117/120	78	*Burkholderia pseudomallei *7894	ZP_02487655.1
*27*	20443	21138	baseplate assembly protein (V)	-	GAGGGCGGccggcaacATG	231	phage baseplate assembly protein	33-261/261	72	*Burkholderia *phage ϕ52237	YP_293735.1
*28*	21304	22080	N-4/N-6 DNA methylase	+	ACGTTGcctcagaaccATG	258	site-specific DNA methyltransferase	34-290/291	78	*Burkholderia pseudomallei *K96243	YP_111089.1
*29*	22060	22527	tail completion protein (S)	-	GAGCAATGGGtggcgtgATG	155	phage virion morphogenesis protein	1-155/155	87	*Burkholderia thailandensis *MSMB43	ZP_02466375.1
*30*	22527	22943	tail completion protein (R)	-	AGACGGccgcccATG	138	bacteriophage tail completion protein R	1-138/138	73	*Burkholderia pseudomallei *K96243	YP_111086.1
*31*	22936	23220	Rz1 (LysC)	-	GGAGActcatcgATG	94	hypothetical phage protein	1-83/91	75	*Burkholderia pseudomallei *1655	ZP_04890536.1
*32*	23057	23497	Rz (LysB)	-	GAAGGcggccgcGTG	146	protein lysB	1-146/146	62	*Burkholderia thailandensis *E264	YP_439538.1
*33*	23494	24303	endolysin	-	GGAGCAccgaatcATG	269	putative phage-encoded peptidoglycan binding protein	1-269/270	73	*Burkholderia pseudomallei *K96243	YP_106791.1
*34*	24300	24572	holin	-	AGGGGGAAAtgacATG	90	protein of unknown function DUF754	1-88/88	67	*Burkholderia sp*. CCGE1001	ZP_06292819.1
*35*	24574	24918	putative antiholin	-	GGAAttgtccgaATG	114	hypothetical protein Bpse38_23639	1-113/114	83	*Burkholderia thailandensis *MSMB43	ZP_02466369.1
*36*	24934	25140	tail protein (X)	-	GGTTGAActgatctgATG	68	phage tail protein X	1-68/68	88	*Burkholderia pseudomallei *7894	ZP_02487665.1
*37*	25140	25619	capsid completion protein (L)	-	GAATCGaccATG	159	fels-2 prophage protein	1-159/159	86	*Burkholderia thailandensis *E264	ZP_05590935.1
*38*	25719	26408	terminase endonuclease subunit (M)	-	GAGCTGGtggcggcATG	229	hypothetical protein bglu_1 g01450	1-228/229	95	*Burkholderia glumae *BGR1	YP_002910051.1
*39*	26405	27418	capsid protein (N)	-	GGAGAAcccaactcATG	337	Gp2, phage major capsid protein, P2 family protein	1-337/337	98	*Burkholderia glumae *BGR1	YP_002910052.1
*40*	27454	28266	capsid scaffolding protein (O)	-	GGTTCGAcctctctctATG	270	phage capsid scaffolding protein (GPO)	1-270/270	95	*Burkholderia glumae *BGR1	YP_002910053.1
*41*	28345	30180	terminase ATPase subunit (P)	+	AACGAGcggcgtATG	611	phage terminase, ATPase subunit	1-589/589	99	*Burkholderia glumae *BGR1	YP_002910054.1
*42*	30177	31226	portal vertex protein (Q)	+	GGAGttctattcATG	349	Gp5, phage portal protein, pbsx family protein	1-347/351	99	*Burkholderia glumae *BGR1	YP_002910055.1
*43*	31595	31732	unknown	+	GATGcgcgATG	45	none				
*44*	31722	33290	unknown	-	GGGGAAAGcaacatATG	522	hypothetical protein ECO103_1901	1-526/527	54	*E. coli *O103:H2 str. 12009	YP_003221840.1
*45*	33455	34699	EcoRII-C endonuclease	-	AACGGAGcttcggggATG	414	type II restriction endonuclease, EcoRII-C domain protein	1-401/401	77	*Candidatus *Hamiltonella defensa 5AT (*Acyrthosiphon pisum*)	YP_002923978.1
*46*	34696	35142	Vsr endonuclease	-	TCGCctgATG	148	DNA mismatch endonuclease Vsr	1-148/148	77	*Burkholderia graminis *C4D1M	ZP_02883050.1
*47*	35142	36410	EcoRII DNA cytosine methylase	-	AGCGAGAGcaaatATG	422	DNA-cytosine methyltransferase	1-426/426	81	*Burkholderia phytofirmans *PsJN	YP_001894783.1
*48*	36570	37685	unknown	-	AAGCTGAcgctATG	372	conserved hypothetical protein	1-371/371	95	*Burkholderia ambifaria *MEX-5	ZP_02905764.1
*49*	37728	38816	unknown	-	AGTTctctaattgacATG	362	GP30 family protein	1-362/362	96	*Burkholderia ambifaria *MEX-5	ZP_02905765.1
*50*	38822	39526	unknown	-	GGGAGAAGcctgaATG	234	VRR-NUC domain protein	1-234/234	99	*Burkholderia ambifaria *MEX-5	ZP_02905766.1
*51*	39523	40032	unknown	-	AGGAGttcagcATG	169	PAAR repeat-containing protein	1-169/169	97	*Burkholderia ambifaria *MEX-5	ZP_02905767.1
*52*	40202	40408	transcriptional regulator	+	AAGGAGAAAtagcATG	68	phage transcriptional regulator, AlpA	1-68/72	94	*Burkholderia ambifaria *MEX-5	ZP_02905768.1

Abbreviations: RBS, ribosome-binding site; aa, amino acid; % ID, percent identity. The P2 proteins that are similar to KL3 proteins based on CoreGenes analysis are shown in brackets in the putative function column.

### Genome characterization

#### KS5

The KS5 genome is 37236 base pairs (bp) in length and encodes 46 proteins (including the transposase of a predicted insertion sequence, discussed below) (Table 1). This genome has a 63.71% G+C content. Forty-three of the start codons are ATG, 2 are GTG and 1 is TTG (Table 1). As KS5 was isolated from an environmental sample, it was predicted that this phage might be obligately lytic [25]. However, KS5 encodes an integrase and a repressor and is found as a prophage in chromosome 2 of the fully sequenced *B. multivorans *strain ATCC 17616 (GenBank:NC_010805.1; BMULJ_03640-BMULJ_03684, bp 477496-514731) (Table 1). Because of this similarity, the possibility exists that KS5 originated from ATCC 17616 or a closely related strain found in the soil enrichment. Excluding the ATCC 17616 prophage, KS5 is most similar to a putative prophage element in *Burkholderia multivorans *CGD1. Twenty-three of 46 KS5 proteins are most closely related to a protein from CGD1, with percent identities ranging from 72-99% (Table 1).

#### KS14

The KS14 genome is 32317 bp in length and encodes 44 proteins (Table 2). This genome has a 62.28% G+C content. Forty-one of the start codons are ATG, 2 are GTG and 1 is TTG (Table 2). All predicted KS14 proteins show similarity to at least one protein in the database (as determined by a BLASTP search) except for gp38 and gp42. The protein with the most similarity to others in the database is the terminase large subunit, gp35, which has 75% identity with a protein of unknown function DUF264 of *Burkholderia sp*. CCGE1001. Aside from gp38 and gp42, the least similar protein is the hypothetical protein gp39, which has 29% identity with the flagellar hook-associated protein FlgK of *Acidovorax ebreus *TPSY (Table 2).

#### KL3

The KL3 genome is 40555 bp in length and encodes 52 proteins (Table 3). This genome has a 63.23% G+C content. Fifty-one of the start codons are ATG and 1 is GTG (Table 3). Similarly to KS14, all predicted KL3 proteins show similarity to at least one protein in the database except for gp43. The proteins with the most similarity to others in the database are the terminase large subunit (gp41) and the portal protein (gp42) that have 99% identity with *Burkholderia glumae *BGR1 proteins and the hypothetical protein gp50 which has 99% identity with a *B. ambifaria *MEX-5 protein. Aside from gp43, the least similar protein is the hypothetical protein gp14, which has 50% identity with the hypothetical protein BuboB_27112 of *Burkholderia ubonensis *Bu (Table 3).

#### Modular organization

**Figure 2:**
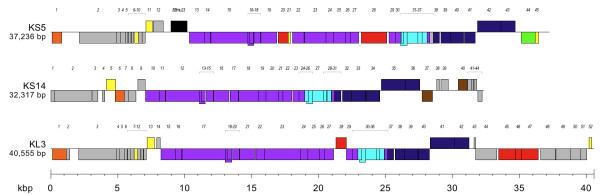
**Genome maps of KS5, KS14 and KL3**. Genes transcribed from the plus strand are shown above and genes transcribed from the minus strand are shown below. The scale (in kbp) is shown on the bottom. The prophage gene order is shown for KS5 and KL3. The gene order for KS14 (which is maintained as a plasmid prophage) was chosen based on alignment with the other two sequences. Legend: orange, recombinase; yellow, transcriptional or translational regulation; black, insertion sequence; purple, tail morphogenesis; red, DNA modification; light blue, lysis; dark blue, capsid morphogenesis and DNA packaging; green, reverse transcription; brown, replication and partitioning; gray, unknown function.

The genome maps of KS5, KS14 and KL3 are shown in Figure 2. Each of these phages has a modular organization, with genes for tail formation (shown in purple), lysis (shown in light blue) and head formation (shown in dark blue) clustered in each phage (Figure 2). In KS5, genes *13-19*, *22-27*, *29-30 *and *36 *encode tail proteins, genes *31-35 *encode lysis proteins and genes *38-43 *encode capsid proteins (Table 1, Figure 2). In KS14, genes *10-24 *and *30 *encode tail proteins, genes *25-29 *encode lysis proteins and genes *31-36 *encode capsid proteins (Table 2, Figure 2). In KL3, genes *15-27*, *29-30 *and *36 *encode tail proteins, genes *31-35 *encode lysis proteins and genes *37-42 *encode capsid proteins (Table 3, Figure 2). In addition, KL3 has a three-gene DNA modification module (discussed below): gene *45*, encoding an EcoRII-C restriction endonuclease, gene *46*, encoding a Vsr endonuclease and gene *47*, encoding an EcoRII methylase (Table 3, Figure 2).

### Similarity to P2

**Figure 3:**
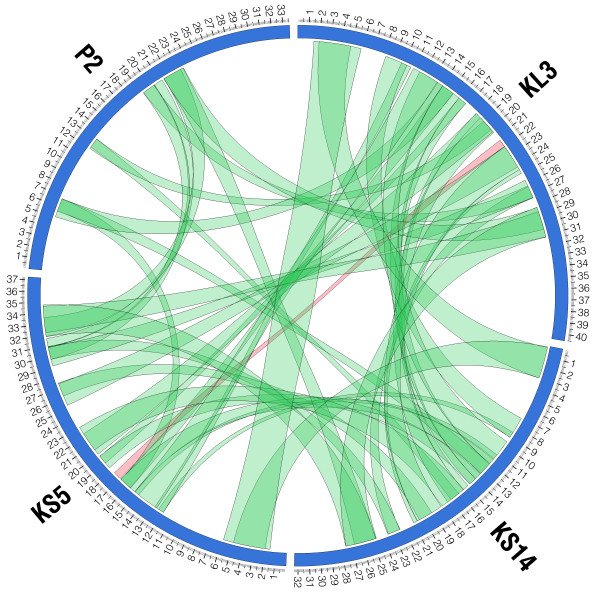
**PROmer/MUMmer/Circos comparison of the KS5, KS14, KL3 and P2 prophages**. Regions of similarity on the same strand are shown in green and regions of similarity on the opposite strand are shown in red. The scale (in kbp) is shown on the outside. The sequence start site for the KS14 prophage (which is maintained as a plasmid) was chosen based on alignment with the other three sequences. PROmer parameters: breaklen = 60, maxgap = 30, mincluster = 20, minmatch = 6.

KS5, KS14 and KL3 all show similarity to enterobacteria phage P2 [GenBank:NC_001895.1]. A four-way comparison of the P2, KS5, KS14 and KL3 genomes prepared using PROmer/MUMmer/Circos is shown in Figure 3. In this comparison, regions of similarity on the same strand are shown in green, while regions of similarity on the opposite strand are shown in red. The majority of similar regions among these phages are on the same strand, except for a short conserved region in KS5 and KL3 containing DNA methylase genes (KS5 *20 *and KL3 *28*, discussed below) on the minus strand in KS5 and on the plus strand in KL3 (Tables 1 and 3). KS5, KS14 and KL3 all encode proteins similar to phage P2 D, U, T, E, E+E', FII, FI, I, J, W, V, S, R and X (involved in tail formation) and L, M, N, O, P and Q (involved in capsid formation) (Table 4). In addition, KS5 gp8 and KL3 gp9 are similar to Ogr (transcriptional activator), KS5 gp28 is similar to Old (phage immunity protein), KS14 gp17 is similar to G (tail fiber assembly protein) and KS14 gp26/gp25 and KL3 gp32/gp31 are similar to LysBC (Rz/Rz1-like lysis proteins, discussed below) (Table 4). The percent identity of the similar proteins ranges from 25-64% in KS5, 24-64% in KS14 and 31-62% in KL3 (Table 4).

**Table 4:** CoreGenes comparison of P2, KS5, KS14 and KL3

P2 protein	P2 function	Similar KS5 protein (% ID)	Similar KS14 protein (% ID)	Similar KL3 protein (% ID)
Old	phage exclusion	gp28 (25%)		
Tin	phage exclusion			
Orf91	unknown			
A	DNA replication			
Orf83	unknown			
Orf82	unknown			
Orf81	unknown			
Orf80	unknown			
B	DNA replication			
Orf78	unknown			
Cox	transcriptional repressor; excision			
C	repressor			
Int	integrase			
Ogr	transcriptional activator	gp8 (39%)		gp9 (34%)
D	tail protein	gp13 (50%)	gp10 (38%)	gp15 (41%)
U	tail protein	gp14 (48%)	gp11 (45%)	gp16 (40%)
T	tape measure protein	gp15 (28%, 42%)	gp12 (25%)	gp17 (31%, 33%)
E	tail protein	gp16 (59%)	gp13 (55%)	gp18 (55%)
E+E'	tail protein	gp17 (50%)	gp14 (49%)	gp19 (51%)
FII	tail tube protein	gp18 (48%)	gp15 (48%)	gp20 (48%)
FI	tail sheath protein	gp19 (64%)	gp16 (64%)	gp21 (58%)
Z/Fun	phage exclusion			
G	tail fiber assembly		gp17 (24%)	
H	tail fiber protein			
I	baseplate assembly protein	gp24 (37%)	gp19 (36%)	gp24 (39%)
J	baseplate assembly protein	gp25 (48%)	gp20 (49%)	gp25 (44%)
W	baseplate assembly protein	gp26 (43%)	gp21 (39%)	gp26 (36%)
V	baseplate assembly protein	gp27 (38%)	gp22 (35%)	gp27 (31%)
Orf30	unknown			
S	tail completion protein	gp29 (44%)	gp23 (35%)	gp29 (35%)
R	tail completion protein	gp30 (43%)	gp24 (43%)	gp30 (39%)
LysC	Rz1-like		gp25 (36%)	gp31 (48%)
LysB	Rz-like		gp26 (33%)	gp32 (42%)
LysA	antiholin			
K	endolysin			
Y	holin			
X	tail protein	gp36 (51%)	gp30 (55%)	gp36 (62%)
L	capsid completion protein	gp38 (45%)	gp31 (39%)	gp37 (43%)
M	terminase small/endonuclease subunit	gp39 (49%)	gp32 (47%)	gp38 (46%)
N	major capsid protein	gp40 (51%)	gp33 (54%)	gp39 (55%)
O	capsid scaffolding protein	gp41 (46%)	gp34 (44%)	gp40 (40%)
P	terminase large/ATPase subunit	gp42 (59%)	gp35 (60%)	gp41 (57%)
Q	portal protein	gp43 (57%)	gp36 (54%)	gp42 (55%)

Abbreviations: % ID, percent identity.

The genes in common between P2 and the P2-like BCC phages are almost exclusively limited to structural genes involved in virion formation (Table 4). Other P2 genes, such as those involved in DNA replication, phage immunity, lysogeny and lysis are dissimilar among these phages. A similar pattern is observed (with some exceptions) following CoreGenes analysis of the P2-like phages ϕE202 of *B. thailandensis *and ϕ52237 and ϕE12-2 of *B. pseudomallei *(data not shown) [23]. A likely explanation for this pattern is that, while phage structural components predominantly interact with each other, components from other phage systems may interact with host-specific proteins (such as those involved in transcription and DNA replication) [31,32]. KS5, KS14 and KL3 appear to have retained P2 modules for the closely interacting capsid and tail proteins, while acquiring new modules for carrying out *Burkholderia *host-specific processes. These genes replace P2 genes at the right end of the P2 genome (the TO-region), P2 *Z*/*fun *(the Z-region) and P2 *orf30 *(Table 4) [33]. As it is very common for genes not found in P2 to be identified in these three regions in other P2-like phages, it is predicted that these loci contain genes that have been acquired via horizontal transfer [33].

Although a phage may show relatedness to a well-characterized phage such as P2, specific guidelines must be used to determine both the degree of relatedness of two phages and if the novel phage can be classified as a "P2-like virus" in a strict taxonomic sense. Lavigne et al. proposed the use of the comparison program CoreGenes to aid in phage taxonomic analysis [34]. This program can be used to compare the proteomes of two or more phages [34]. If a phage shares at least 40% of its proteins (those with a BLASTP score ≥ 75) with a reference phage such as P2, then these two phages can be considered as part of the same genus, while if it shares 20-39% of its proteins with a reference phage, then they can be considered as part of the same subfamily [34]. When KS5, KS14 and KL3 were analyzed with CoreGenes using P2 as a reference genome, the percentage of proteins in common with respect to P2 were 51.16%, 53.49% and 53.49%, respectively. These are similar to the percentages for ϕE202 (55.81%), ϕ52237 (51.16%) and ϕE12-2 (48.84%) [23]. Based on these results, KS5, KS14 and KL3 can be classified as members of the *Peduovirinae *subfamily and "P2-like viruses" genus [23].

### Integration site characterization

In *E. coli*, P2 is able to integrate at over 10 different loci, but certain sites may be used more commonly than others [35]. None of the three P2-like BCC phages characterized here were found to integrate into a locus similar to that of P2. Only KL3 was found to have a previously characterized integration site. Following PCR amplification and sequencing from the *B. cenocepacia *CEP511 chromosome (where KL3 is carried as a prophage), it was determined that, like many phages, KL3 integrates into a tRNA gene. Specifically, it integrates into the middle of a threonine tRNA gene: bp 1 of the KL3 prophage corresponds to bp 32 of the tRNA based on comparison with a 76 bp threonine tRNA gene of *B. cenocepacia *HI2424 chromosome 1 (Bcen2424_R0015, bp 491047-491122). Other phages that integrate into threonine tRNA genes include enterobacteria phage P22, *Shigella flexneri *phage SfV and *Salmonella enterica *serovar Typhimurium phage ST104 [36-38]. KL3 integration should not affect threonine tRNA synthesis as bp 1-45 of KL3 has an identical sequence to bp 32-76 of the tRNA gene.

In both *B. multivorans *ATCC 17616 and *B. cenocepacia *C6433, KS5 integrates into the 3' end of an AMP nucleosidase gene. AMP nucleosidases convert AMP into adenine and ribose 5-phosphate [39]. This gene has not been previously identified as a phage integration site. KS5 bases 1-815 (including the integration site and the integrase gene sequence) show similarity to sequences encoding pairs of adjacent AMP nucleosidase and integrase genes in several *Burkholderia *genomes. For example, in *B. pseudomallei *K96243 chromosome 2, the AMP nucleosidase (BPSS1777) and integrase (BPSS1776) genes are adjacent to genes annotated as encoding a putative phage capsid related protein (fragment) (BPSS1775) and putative phage-related tail protein (fragment) (BPSS1774A). Similarly, in *B. pseudomallei *1106a chromosome 2, the AMP nucleosidase (BURPS1106A_A2416) and integrase (BURPS1106A_A2415) genes are adjacent to genes annotated as encoding a phage portal domain protein (BURPS1106A_A2414) and phage tail completion protein (BURPS1106A_A2413). The identification of phage related genes at this site in other *Burkholderia *genomes suggests that the AMP nucleosidase gene may be a conserved integration site among some *Burkholderia*-specific temperate phages.

**Figure 4:**
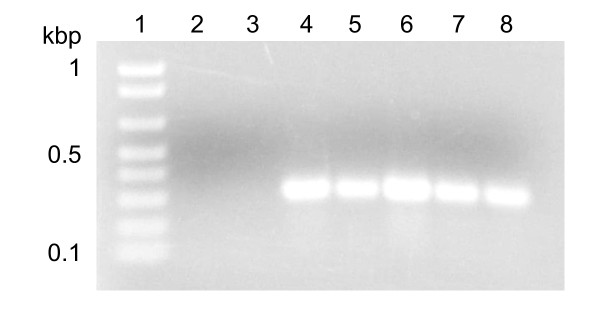
**Detection of lysogeny in KS14-resistant *B. cenocepacia *C6433 isolates **[19]. Bacterial genomic DNA was amplified using KS14-specific primers. Lane 1: 1 Kb Plus DNA ladder (Invitrogen), lane 2: DNA-free control, lane 3: C6433 control, lane 4: KS14-resistant C6433 isolate I, lane 5: KS14-resistant C6433 isolate II, lane 6: KS14-resistant C6433 isolate III, lane 7: KS14-resistant C6433 isolate IV, lane 8: KS14-resistant C6433 isolate V. The size of the markers (in kbp) is shown on the left.

KS14 is different from other P2-like phages in that it does not encode a tyrosine integrase. Most temperate phages use a tyrosine recombinase (or, in rare cases, a serine recombinase) to facilitate recombination between the phage *attP *site and the host *attB *site [40]. KS14 encodes a serine recombinase (gp6), but this protein is unlikely to mediate prophage integration for three reasons. First, gp6 is more closely related to invertases such as Mu Gin (49% identity, E-value: 8e^-44^) and P1 Cin (49% identity, E-value: 7e^-43^) than to integrases such as those from *Streptomyces lividans *phage ϕC31 (29% identity, E-value: 1.2) and *Mycobacterium smegmatis *phage Bxb1 (29% identity, E-value: 3e^-4^) [41-44]. Second, gp6 lacks the conserved cysteine-rich and leucine/isoleucine/valine/methionine-rich regions found in other serine integrases [45]. Third, gp6 is only 225 aa in length, which is substantially smaller than the serine integrases that are typically between 450-600 aa in length [45]. We did not believe KS14 to be obligately lytic because it encodes a putative repressor protein (gp5) and because previously collected KS14-resistant C6433 isolates were predicted to be lysogenized based on PCR-positivity with KS14-specific primers (Figure 4) [19].

**Figure 5:**
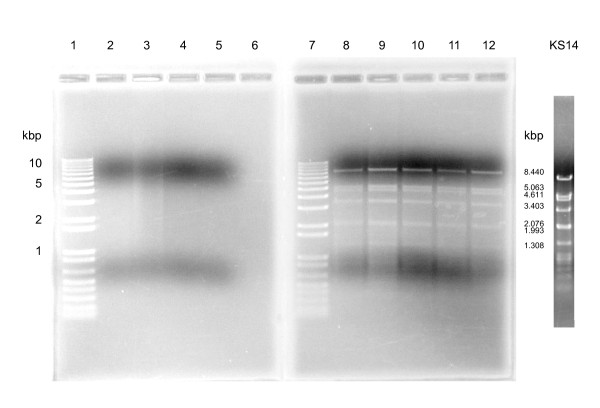
**Isolation of the KS14 plasmid prophage**. DNA was isolated using a QIAprep Spin Miniprep plasmid isolation kit (Qiagen) and digested with EcoRI (Invitrogen). Lane 1: 1 Kb Plus DNA ladder (Invitrogen), lane 2: *B. cenocepacia *C6433, lane 3: *B. multivorans *ATCC 17616, lane 4: *B. cenocepacia *CEP511 lane 5: *B. cenocepacia *K56-2, lane 6: blank, lane 7: 1 Kb Plus DNA ladder, lane 8: KS14-resistant C6433 isolate I, lane 9: KS14-resistant C6433 isolate II, lane 10: KS14-resistant C6433 isolate III, lane 11: KS14-resistant C6433 isolate IV, lane 12: KS14-resistant C6433 isolate V. The size of the markers (in kbp) is shown on the left. A KS14 EcoRI DNA digest and the size of the bands predicted for this digest (> 1 kbp in size) are shown on the far right.

Phages such as P1, P7 and N15 of enterobacteria, ϕ20 of *Bacillus anthracis*, ϕBB-1 of *Borrelia burgdorferi*, LE1 of *Leptospira biflexa*, pGIL01 of *Bacillus thuringiensis *and pKO2 of *Klebsiella oxytoca *lysogenize their hosts as plasmids [46-53]. Because KS14 gene *39 *encodes a putative ParA protein (involved in partitioning in other plasmid prophages), we predicted that the KS14 prophage might exist as a plasmid [54,55]. To test this hypothesis, we used a standard protocol for the QIAprep Spin Miniprep plasmid isolation kit with cells of C6433 (a KS14 host), ATCC 17616 (a KS5 lysogen), CEP511 (a KL3 lysogen), K56-2 (a lysogen of KS10, a previously characterized BCC-specific phage) and five putatively lysogenized KS14-resistant C6433 isolates [19,56]. These preparations were then treated with EcoRI and the resulting fragments were separated using agarose gel electrophoresis. For each of the four control strains, no distinct bands were observed (Figure 5, left). In contrast, preparations from each of the five putatively lysogenized strains contained identical distinct bands (Figure 5, right). Furthermore, these bands were the same size as those predicted and observed for an EcoRI digest of KS14 DNA (with predictions based on a circular genome sequence) (Figure 5, far right) and sequences from selected bands matched the KS14 genome sequence. Based on these results, we predict that KS14 is a temperate phage that, in contrast to other P2-like phages, lysogenizes host strains as a plasmid.

It is important to note that, although one of these phages has been shown to be active *in vivo*, temperate phages are generally considered to be suboptimal for use in a phage therapy protocol [19,21]. In contrast to obligately lytic phages, temperate phages are associated with superinfection immunity, lysogenic conversion and specialized transduction [reviewed in] [21]. In a previous study, we have shown that temperate BCC-specific phages can be engineered to their lytic form by inactivating the repressor gene [21]. This strategy could potentially be used with the three phages described here, thus making them more appropriate candidates for clinical use.

### Morphogenesis genes

As discussed above, the KS5, KS14 and KL3 structural genes are related to those from P2 and function to construct a P2-like myovirus with a contractile tail. The only virion morphogenesis genes of P2 that these phages lack are *G *(encoding the tail fiber assembly protein, missing in KS5 and KL3) and *H *(encoding the tail fiber protein) (Table 4). Because the tail fibers are involved in host recognition, it is expected that these proteins would be dissimilar in phages infecting *E. coli *and those infecting the BCC.

**Figure 6:**
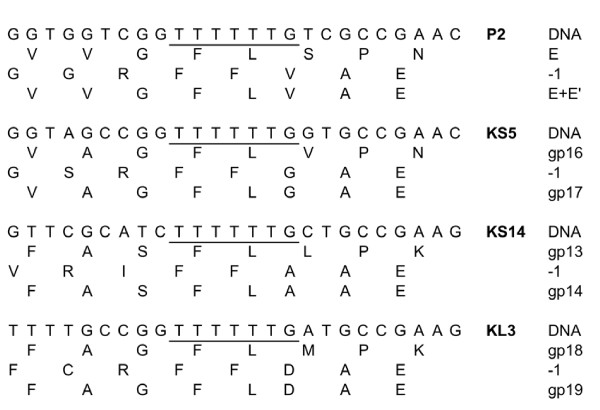
**Conservation of the P2 *E*/*E+E' *frameshift sequence in KS5, KS14 and KL3**. For each phage, the DNA sequence is shown in the first line, the translation in the original frame is shown in the second line, the translation in the -1 frame is shown in the third line and the amino acid sequence of the frameshifted protein is shown in the fourth line. The conserved TTTTTTG frameshift sequence is underlined. The frameshift is predicted to occur after the terminal G in this sequence.

A commonly identified characteristic in tailed phages is the expression of two tail proteins from a single start codon via a translational frameshift [57]. These proteins (encoded in a region between the genes for the tail tape measure and the major tail protein) share the same N-terminus but have different C-termini due to stop codon readthrough in the -1 frame [57]. In P2, this -1 frameshift occurs at a TTTTTTG sequence and produces the 91 aa protein E and the 142 aa protein E+E' from the same translational start site (Figure 6) [57,58]. KS5, KS14 and KL3 all encode proteins similar to both E and E+E' with percent identities ranging from 49-59% (Table 4). Despite the relatively low degree of similarity, the P2 frameshift site appears to be conserved amongst these phages, suggesting that they likely use a similar frameshifting mechanism (Figure 6). In rare cases, RNA secondary structure can be identified downstream of the phage frameshift sequence [21,57]. When the KS5, KS14 and KL3 *E+E' *sequences 60 bp downstream of the TTTTTTG sequence were screened for secondary structure, no predicted hairpins were identified (data not shown). This result was anticipated based upon the absence of these structures in the P2 phage *E+E' *gene [57].

### Lysis genes

In P2, the lysis module consists of five genes: *Y *(holin), *K *(endolysin), *lysA *(antiholin), *lysB *(Rz) and *lysC *(Rz1) [59,60]. The P2-like BCC phages are predicted to encode endolysins, holins and antiholins that are dissimilar to those of P2 (Table 4). KS5 gp33, KS14 gp27 and KL3 gp33 are putative endolysins as they all have the conserved domain pfam01471 (PG_binding_1, putative peptidoglycan binding domain; E-values: 3e^-11^, 3e^-10 ^and 9e^-10^, respectively) and show similarity to other phage endolysins. P2 Y is a type I holin with three transmembrane domains [61]. Although KS5 *34*, KS14 *28 *and KL3 *34 *are dissimilar to P2 *Y*, it is predicted that these three genes encode holins because they are each immediately upstream of a putative endolysin gene and they each encode proteins that a) have three transmembrane domains based on OCTOPUS analysis and b) show similarity to other phage holins.

Antiholins such as P2 LysA inhibit holin activity and delay lysis of infected cells in order to optimize the phage burst size [59,62]. Although some phages such as λ express antiholins from a second translational start site two codons upstream of the holin start codon, phages such as P2 and ϕO1205 of *Streptococcus thermophilus *encode an antiholin from a separate gene [63,59,64]. The location of the putative antiholin genes KS5 *35*, KS14 *29 *and KL3 *35 *is similar to that in ϕO1205, in which the holin and antiholin genes are adjacent immediately upstream of the endolysin gene (as opposed to P2, in which gene *K *separates *Y *and *lysA*) [64,59]. Based on OCTOPUS analysis, KS5 gp35 has three transmembrane domains, while KS14 gp29, KL3 gp35 and P2 LysA have four. Based on gene organization and protein transmembrane structure, it is predicted that the P2-like BCC phages have separate antiholin genes in their lysis modules.

**Figure 7:**
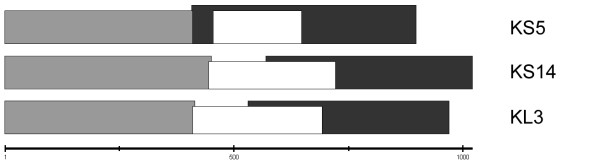
**Organization of the *lysBC *genes in KS5, KS14 and KL3**. *R *genes (KS5 *30*, KS14 *24 *and KL3 *30*) are shown in light gray, *lysB *genes (KS5 *32*, KS14 *26 *and KL3 *32*) are shown in dark gray and *lysC *genes (KS5 *31*, KS14 *25 *and KL3 *31*) are shown in white. The scale (in bp) is shown below.

P2 encodes two proteins, LysB and LysC, that are predicted to function similarly to λ Rz and Rz1 [60]. Rz is an inner membrane protein with an N-terminal transmembrane domain and Rz1 is a proline-rich outer membrane lipoprotein [65]. Rz/Rz1 pairs fuse the inner and outer membranes following holin and endolysin activity and facilitate phage release [65]. The P2 *lysC *start codon is in the +1 frame within the *lysB *gene, while the *lysC *stop codon is out of frame in the downstream tail gene *R *[66]. In contrast, the *Rz1 *gene in λ is entirely contained within the *Rz *gene [67]. KS14 and KL3 LysBC pairs (gp26/gp25 and gp32/gp31, respectively) are similar to that of P2 (Table 4). In KS14 and KL3, the *lysC *genes start approximately 160 bp upstream from the *lysB *stop codon and extend into the first 8 bp of *R *(gene *24 *in KS14 and *30 *in KL3) (Figure 7). Both KS14 and KL3 LysC proteins are predicted to have a signal peptidase II cleavage site between positions 20 (alanine) and 21 (cysteine). Signal peptidase II cleavage would produce a 72 aa lipoprotein with 7 prolines (9.7% proline) for KS14 LysC and a 74 amino acid lipoprotein with 7 prolines (9.5% proline) for KL3 LysC.

In contrast to the P2-like *lysBC *gene organization found in KS14 and KL3, the KS5 genes *32*/*31 *have a similar organization to λ *Rz*/*Rz1*. KS5 Rz1 is encoded in the +1 frame within the *Rz *gene (Figure 7). It is predicted to have a signal peptidase II cleavage site between positions 18 (alanine) and 19 (cysteine), which would produce a 46 amino acid lipoprotein with 12 prolines (26.1%). The differences in both gene organization and proline content between the P2-like KS14 and KL3 LysC proteins and the λ-like KS5 Rz1 protein suggest that KS5 may have acquired genes *31 *and *32 *- and potentially the entire lysis module - through horizontal transfer from a phage similar to λ.

### Sequence elements unique to KS5 and/or KL3

#### Insertion sequences

Insertion sequences (ISs) are short genetic elements that can insert into nonhomologous regions of DNA [68]. These elements, comprised of a transposase gene and inverted repeats, create flanking direct repeats following insertion [68]. Many mutants of well-characterized phages have been found to carry ISs, including λ and Mu [69,70]. However, it is relatively rare for wildtype phages to carry ISs because they can interfere with gene expression [71]. Sakaguchi et al. determined the genome sequence of the *Clostridium botulinum *phage c-st and determined that it carries 12 ISs (5 of which are incomplete) [71]. Of the 284 genomes sequenced at the time, one IS was found in each of eight phages: *Burkholderia *phages ϕE125 and Bcep22, enterobacteria phages P1 and HK022, *Lactobacillus *phages ϕAT3 and LP65, *Rhodothermus *phage RM378 and *Shigella *phage Sf6 [71].

**Figure 8:**
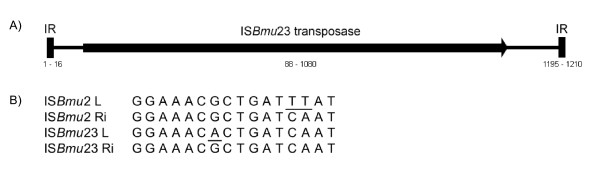
**Comparison of the IS*Bmu*23 and IS*Bmu*2 insertion sequences**. A) Structure of IS*Bmu*23. IR, inverted repeats. Relative positions of the inverted repeats and transposase gene (in bp) are shown below. B) Alignment of the IS*Bmu*2 and IS*Bmu*23 inverted repeats. Non-consensus bases are underlined. IS*Bmu*2 sequences are from Ohtsubo et al. [72]. L, left repeat; Ri, right repeat inverted.

A novel insertion sequence (named IS*Bmu*23 in vB_BmuZ-ATCC 17616) is found in the KS5 genome between gene *12*, encoding a membrane protein and gene *13*, encoding the tail protein D (Table 1). This IS does not appear to disrupt any putative ORFs and so may not have any significant effect on phage gene expression. IS*Bmu*23 is 1210 bp in length and contains two imperfect 16 bp inverted repeats (Table 1, Figure 8). In KS5, it is flanked by two copies of a 5 bp direct repeat, CCTAA. IS*Bmu*23 encodes a 330 aa transposase that has the conserved domain COG3039 (transposase and inactivated derivatives, IS5 family; E-value: 8e^-29^). This protein is most similar to the transposase of IS*Bmu*2 (85% identity), an IS5-like IS present in nine copies in ATCC 17616 [72]. IS*Bmu*2 and IS*Bmu*23 are very similar as they a) are present in the same genome, b) are both 1210 bp in length, c) encode similar 330 aa transposases, d) have similar 16 bp inverted repeats (the right inverted repeats of IS*Bmu*2 and IS*Bmu*23 are identical, while the left repeats differ by 3 bp) and e) preferentially integrate into CTAA sequences (Figure 8). Ohtsubo et al. found that the transposition of ISs in ATCC 17616 increased when the cells were grown at high temperatures [72]. Because these temperatures are similar to what the cell may encounter during infection of an animal or human, it is suggested that this change may provide a selective advantage to ATCC 17616 by modifying its genome under *in vivo *conditions [72]. Further experiments are required to determine if IS*Bmu*23 transposition is affected by temperature and if this IS may provide a selective advantage to KS5 lysogens *in vivo*.

#### Reverse transcriptases

Reverse transcriptases (RTs) are RNA-dependent DNA polymerases most commonly associated with retroviruses and retrotransposons [73]. RTs have also been identified in several phage genomes, including those of P2-like phages [74-76]. One function of these proteins was extensively characterized in *Bordetella bronchiseptica *phage BPP-1. This phage has the ability to change its host range by making amino acid substitutions in its tail fiber protein, Mtd (major tropism determinant) [77]. This switch requires the phage-encoded RT Brt (*Bordetella *RT) that synthesizes a DNA copy of a 134 bp locus (the template repeat, TR) that has 90% identity with a 134 bp region of the *mtd *gene (the variable repeat, VR) [77,74]. Adenines in the reverse transcribed copy of TR are mutagenized and the altered DNA integrates or recombines at VR by an unknown mechanism, generating a tail fiber gene with multiple base substitutions [74,75].

A second function associated with phage RTs is phage exclusion. In *Lactococcus lactis*, expression of the putative RT AbiK lowers the efficiency of plating of infecting phages by an unknown mechanism (potentially involving single-strand annealing recombinases) [78]. Expression of Orf570, an RT identified in the P2-like enterobacteria prophage P2-EC30, was found to inhibit T5 infection of *E. coli *[76]. When a region of Orf570 containing an RT conserved motif was deleted, T5 infection was no longer inhibited [76].

KS5 encodes a putative RT, gp44. This protein has the conserved domain cd03487 (RT_Bac_retron_II, reverse transcriptases in bacterial retrotransposons or retrons; E-value: 2e^-45^). It is unlikely that gp44 and Brt have the same function: the two proteins show minimal similarity (21% identity, E-value: 7e^-4^), gene *44 *is located distal to the tail fiber gene (in contrast to *brt *and *mtd*), neither nucleotide substitutions in the tail fiber gene nor variations in KS5 tropism were observed and no repeated sequences were identified in the KS5 genome longer than 28 bp [77]. When compared to Orf570, gp44 shows almost no relatedness (41% over 12/546 amino acids; E-value: 2.7) but is found at the same locus (in the prophage, both *orf570 *and *44 *would be located proximal to the portal protein gene *Q*). Further experiments are required to determine if the KS5 RT is involved in tropism modification, phage exclusion or some uncharacterized function.

#### DNA methylation, restriction and repair

DNA methylase and endonuclease genes are commonly found in phage genomes. Methylases modify the DNA such that it becomes resistant to bacterial restriction systems [79]. Although P2 does not encode any putative methylases, such proteins are encoded by both KS5 and KL3 (KS5 gp20 and KL3 gp28 and gp47) (Tables 1 and 3). All three methylases are predicted to belong to the AdoMet_MTase superfamily (cl12011; S-adenosylmethionine-dependent methyltransferases). KS5 gp20 is most similar to a DNA methylase N-4/N-6 domain protein of *B. ambifaria *MEX-5 (89% identity). KL3 gp28 is most similar to a site-specific DNA methyltransferase of *B. pseudomallei *K96243 (78% identity). Both of these proteins have the conserved domain pfam01555 (N6_N4_Mtase, DNA methylase; KS5 gp20 E-value: 5e^-22^, KL3 gp28 E-value: 4e^-25^). Because this domain is associated with both N-4 cytosine and N-6 adenine methylases, these proteins may have either cytosine or adenine methylase activity [80]. KL3 gp47 shows similarity to a modification methylase EcoRII from several bacterial species, with E-values as low as 4e^-114^. This protein has the conserved domain cd00315 (Cyt_C5_DNA_methylase, Cytosine-C5 specific DNA methylases; E-value: 6e^-68^) and so can be classified as a cytosine-C5 methylase. KS5 gp20 and KL3 gp28 are likely involved in protecting the phage DNA from BCC restriction systems. As discussed below, the function of KL3 gp47 is likely to protect the phage DNA from a phage-encoded restriction enzyme.

Phage nucleases have a number of functions, including degradation of the bacterial DNA (to both inhibit the host and provide nucleotides for the phage), phage exclusion and DNA processing [81]. KL3 encodes two endonucleases, gp45 and gp46. Gp45 is most similar to a type II restriction endonuclease, EcoRII-C domain protein of *Candidatus *Hamiltonella defensa 5AT (*Acyrthosiphon pisum*) (77% identity). This protein has the conserved domain pfam09019 (EcoRII-C, EcoRII C terminal; E-value: 6e^-65^). Gp46 is most similar to a DNA mismatch endonuclease Vsr of *Burkholderia graminis *C4D1M (77% identity). This protein has the conserved domain cd00221 (Vsr, Very Short Patch Repair [Vsr] endonuclease; E-value: 9e^-38^).

The organization of genes *45-47 *(encoding an EcoRII-C endonuclease, Vsr endonuclease and EcoRII methylase, respectively) in a single module suggests that the proteins that they encode are functionally related. The EcoRII-C endonuclease (which has a CCWGG recognition sequence where W = A or T) is likely to degrade either bacterial DNA to inhibit the host during the KL3 lytic cycle or superinfecting phage DNA [81,82]. KL3 DNA would be protected from this cleavage by EcoRII methylation at the second position in the EcoRII-C recognition sequence (forming CC^m^WGG where C^m ^= 5-methylcytosine) [83]. Expression of the Dcm methylase, which has an identical recognition sequence and methylation site as EcoRII methylase, is mutagenic in *E. coli *because 5-methylcytosines are deaminated to thymines, causing T/G mismatches [84,85]. EcoRII methylase expression would presumably cause mismatched sites in KL3 with the sequence C(T/G)WGG. In *E. coli*, these mismatches are repaired by very short patch (VSP) repair which starts with the recognition and nicking of the sequence C(T/G)WGG by a Vsr endonuclease [86]. As KL3 expresses a Vsr endonuclease, it could repair post-methylation T/G mismatches using the same mechanism.

The proposed model for methylase and endonuclease interaction during the KL3 lytic cycle is as follows. Unmethylated host DNA (or, alternatively, superinfecting phage DNA) is degraded by gp45. KL3 DNA is protected from gp45 degradation by gp47-mediated conversion of cytosine to 5-methylcytosine. These 5-methylcytosine bases are deaminated to thymine, but the resulting T/G mismatches are cleaved by gp46 and fixed using VSP repair. Although further experiments are required to test the validity of this model, KL3 appears to encode an elegant system for degradation of bacterial or superinfecting phage DNA, protection of the phage genome and repair of resulting mutations.

## Conclusions

This study is the first to identify and characterize P2-like phages of the BCC. Like other previously characterized P2-like *Burkholderia *phages, KS5, KS14 and KL3 share structural genes with P2 but encode dissimilar accessory proteins. KS5, a 37236 bp prophage of *B. multivorans *ATCC 17616, integrates into an AMP nucleosidase gene, has a λ-like *Rz/Rz1 *cassette, carries an IS*Bmu*2-like insertion sequence and encodes a reverse transcriptase. KS14, a 32317 bp phage previously shown to be active against *B. cenocepacia in vivo*, encodes a serine recombinase but is maintained as a plasmid prophage [19]. KL3, a 40555 bp prophage of *B. cenocepacia *CEP511, integrates into a threonine tRNA gene and encodes a series of proteins capable of degrading bacterial or superinfecting phage DNA, methylating the phage genome and repairing methylation-induced mismatches. As KS14 has already been shown to be active *in vivo*, characterization of these three related phages is an important preliminary step in the development of a phage therapy protocol for the BCC.

## Methods

### Bacterial strains and growth conditions

BCC strains used for phage isolation and propagation were obtained from Belgium Coordinated Collection of Microorganisms LMG Bacteria Collection (Ghent, Belgium) and the Canadian *Burkholderia cepacia *complex Research and Referral Repository (Vancouver, BC). Many of the strains used are from the *Burkholderia cepacia *complex experimental strain panel and updated experimental strain panel [29,87]. Strains were grown aerobically overnight at 30°C on half-strength Luria-Bertani (LB) solid medium or in half-strength LB broth with shaking. Transformations were performed with chemically-competent DH5α (Invitrogen, Carlsbad, CA), plated on LB solid medium containing 100 μg/ml ampicillin and grown aerobically overnight at 37°C. Strains were stored in LB broth containing 20% glycerol at -80°C.

### Electron microscopy

To prepare samples for transmission electron microscopy, phage lysates were filter sterilized using a Millex-HA 0.45 μm syringe driven filter unit (Millipore, Billerica, MA), incubated on a carbon-coated copper grid 5 minutes at room temperature and stained with 2% phosphotungstic acid for 2 minutes. Micrographs were taken with the assistance of the University of Alberta Department of Biological Sciences Advanced Microscopy Facility using a Philips/FEI (Morgagni) transmission electron microscope with charge-coupled device camera at 140,000-fold magnification.

### Phage isolation, propagation and DNA isolation

Isolation of KS5 from onion soil and KS14 from *Dracaena* sp. soil has been described previously [25,19]. KL3 was isolated from a single plaque on a lawn of *B. cenocepacia *CEP511. The plaque was isolated using a sterile Pasteur pipette, suspended in 1 ml of suspension medium (50 mM Tris-HCl [pH 7.5], 100 mM NaCl, 10 mM MgSO_4_, 0.01% gelatin solution) with 20 μl CHCl_3 _and incubated 1 hour at room temperature to generate a KL3 stock. KL3 was propagated on *B. ambifaria *LMG 17828 in soft agar overlays: 100 μl of phage stock and 100 μl of liquid culture were incubated 20 minutes at room temperature and 3 ml of soft nutrient agar was added to this mixture, poured onto half-strength LB solid medium and incubated overnight at 30°C.

Phage genomic DNA was isolated using a modified version of a λ proteinase K/SDS lysis protocol [88]. Half-strength LB agarose plates (prepared with soft nutrient agarose) showing confluent phage lysis were overlaid with 3 ml of suspension media and incubated for 6 hours at 4°C on a platform rocker. The lysate was pelleted by centrifugation at 10 000 × g for 2 minutes and filter-sterilized using a 0.45 μm filter. 10 ml of lysate was treated with 10 μl DNase I/10 μl DNase I buffer and 6 μl RNase I (Fermentas, Burlington, ON) and incubated 1 hour at 37°C. Following addition of 0.5 M EDTA (pH 8.0) to 20 mM, proteinase K to 50 μg/ml and SDS to 0.5%, the solution was mixed and incubated 1 hour at 37°C. Standard phenol:chloroform extraction and ethanol precipitation were then used to purify the phage DNA. Samples were resuspended in TE (pH 8.0) and quantified using a NanoDrop ND-1000 spectrophotometer (Thermo Scientific, Waltham, MA).

KS14 plasmid prophage DNA was isolated from five putatively lysogenized KS14-resistant C6433 isolates [19] using a QIAprep Spin Miniprep kit (Qiagen, Hilden, Germany). Lysogeny was predicted using PCR with KS14-specifc primers (KS14F: GCAGCTAACCGAGTCGCACG, KS14R: CTCTGAAAAGGTGGGCGGTGG) (Sigma-Genosys, Oakville, ON) and TopTaq DNA polymerase and buffers (Qiagen). *B. multivorans *ATCC 17616 and *B. cenocepacia *C6433, CEP511 and K56-2 were used as negative controls. 2 ml aliquots of 16 hour overnight cultures (OD_600_: 2.0-2.2) were pelleted, washed 3× with sterile H_2_O to remove exogenous phages and treated using the standard kit protocol. For each sample, 2 20 μl EcoRI (Invitrogen) reactions each containing 17 μl of plasmid DNA were digested overnight, pooled and separated on 0.8% (wt/vol) agarose gels in 1× TAE (pH 8.0).

### Sequencing and bioinformatics analysis

Preliminary sequence analysis was performed using a shotgun cloning protocol. Phage DNA was digested using EcoRI (Invitrogen), separated on 0.8% (wt/vol) agarose gels, purified using the GeneClean II kit (Qbiogene, Irvine, CA), ligated into pUC19 or pGEM-7Z and transformed into DH5α (Invitrogen). Following blue-white selection on LB solid medium containing 100 μg/ml ampicillin, constructs with phage DNA inserts were isolated using a QIAprep Spin Miniprep kit (Qiagen), digested using EcoRI and viewed using gel electrophoresis. Inserts were sequenced with the assistance of the University of Alberta Department of Biological Sciences Molecular Biology Service Unit using an ABI 3730 DNA analyzer (Applied Biosystems, Foster City, CA). Sequences were edited using EditView and aligned using AutoAssembler (Perkin-Elmer, Waltham, MA). For completion of the three genomes, DNA samples were submitted for pyrosequencing analysis (454 Life Sciences, Branford, CT). Gaps between the assembled sequences were filled following PCR amplification and cloning using primers (Sigma-Genosys) designed to amplify across the gaps, TopTaq DNA polymerase and buffers (Qiagen) and the CloneJET PCR cloning kit (Fermentas). The complete genome sequences of KS5, KS14 and KL3 were deposited in GenBank with the accession numbers GU911303, HM461982 and GU911304, respectively.

Annotation of the assembled sequences was performed using GeneMark.hmm-P http://exon.biology.gatech.edu[89]. For KS5, annotations were based on those of the ATCC 17616 chromosome 2 sequence (GenBank:NC_010805.1; BMULJ_03640 - BMULJ_03684, bp 477496-514731). Manual annotations were performed for the *E+E' *and *lysC/Rz1 *genes. Proteins were numbered based on the order of the genes in the prophage (i.e. the integrase gene was named *1 *and the integrase was named gp1). Relatedness of the predicted proteins was assessed using BLASTP http://blast.ncbi.nlm.nih.gov[90]. Protein transmembrane domains, stem-loop structures and signal peptide cleavage sites were identified using OCTOPUS http://octopus.cbr.su.se, mfold http://mfold.rna.albany.edu and LipoP http://www.cbs.dtu.dk/services/LipoP, respectively [91-93]. Repeat sequences in the DNA were identified using REPuter http://bibiserv.techfak.uni-bielefeld.de/reputer[94]. Restriction sites were predicted using NEBcutter http://tools.neb.com/NEBcutter2[95]. Whole genome sequence comparisons were performed using CoreGenes with a stringency setting of 75 http://www.binf.gmu.edu/genometools.html[96,34]. Comparison figures were constructed using PROmer/MUMmer http://mummer.sourceforge.net Circos http://mkweb.bcgsc.ca/circos[97,98].

To identify the KS5 prophage insertion site in ATCC 17616, the assembled KS5 sequence was compared to the vB_BmuZ-ATCC 17616 sequence in a BLASTN search and the left prophage junction was determined. Primers designed to this region (KS5*attL*F: TGCACGGCGAGCTGAAACTG, KS5*attL*R: GAAGGCACGCGAGGTAGAACG) were used to amplify the C6433/KS5 prophage junction in C6433 lysogens. To identify the KL3 insertion site in CEP511, the region proximal to the KL3 integrase gene *1 *was analyzed using BLASTN and found to be similar to a region containing a tRNA-Thr gene in several *Burkholderia *strains including *B. ambifaria *AMMD (Bamb_R0016; chromosome 1, bp 403358-403433). Primers designed to this region (KL3*attL*F: AGCTGCAGATGGGTAACGAGTGG, KL3*attL*R: CCACTCACGAAGGGCAAGCTG) were used to amplify the CEP511/KL3 prophage junction.

## Authors' contributions

KHL isolated KL3, took electron micrographs, isolated, sequenced, annotated and analyzed the three genomes and drafted the manuscript. PS prepared the PROmer/MUMmer/Circos genome comparison. JJD devised the study, assisted with experimental design and interpretation of data, performed sequence data collection and edited the manuscript. All authors have read and approved the final manuscript.
